# Case Report: Favorable outcome of allogeneic hematopoietic stem cell transplantation in SARSCoV2 positive recipient, risk-benefit balance between infection and leukemia

**DOI:** 10.3389/fimmu.2023.1184956

**Published:** 2023-05-23

**Authors:** Chiara Oltolini, Andrea Acerbis, Giorgio Orofino, Sara Racca, Maddalena Noviello, Stefania Dispinseri, Nicola Clementi, Simona Piemontese, Elisabetta Xue, Fabio Giglio, Maria Teresa Lupo Stanghellini, Elisa Diral, Alessandro Bruno, Elena Tassi, Valeria Beretta, Ilaria Marzinotto, Gabriella Scarlatti, Vito Lampasona, Anna Ardemagni, Michela Sampaolo, Chiara Bonini, Consuelo Corti, Jacopo Peccatori, Antonella Castagna, Fabio Ciceri, Raffaella Greco

**Affiliations:** ^1^ Clinic of Infectious Diseases, Istituto di Ricovero e Cura a Carattere Scientifico (IRCCS) San Raffaele Scientific Institute, Milan, Italy; ^2^ Hematology and Bone Marrow Transplantation, Istituto di Ricovero e Cura a Carattere Scientifico (IRCCS) San Raffaele Scientific Institute, Milan, Italy; ^3^ University Vita-Salute San Raffaele, Milan, Italy; ^4^ Laboratory of Microbiology and Virology, Istituto di Ricovero e Cura a Carattere Scientifico (IRCCS) San Raffaele Scientific Institute, Milan, Italy; ^5^ Experimental Haematology Unit, Division of Immunology, Transplantation and Infectious Diseases, Istituto di Ricovero e Cura a Carattere Scientifico (IRCCS) San Raffaele Scientific Institute, Milan, Italy; ^6^ Cell Therapy Immunomonitoring Laboratory (MITiCi), Division of Immunology, Transplantation and Infectious Diseases, Istituto di Ricovero e Cura a Carattere Scientifico (IRCCS) San Raffaele Scientific Institute, Milan, Italy; ^7^ Viral Evolution and Transmission Unit, Istituto di Ricovero e Cura a Carattere Scientifico (IRCCS) San Raffaele Scientific Institute, Milan, Italy; ^8^ Diabetes Research Institute Unit, Istituto di Ricovero e Cura a Carattere Scientifico (IRCCS) San Raffaele Scientific Institute, Milan, Italy

**Keywords:** allogeneic transplant, COVID – 19, SARS – CoV – 2, veno-occlusive disease, acute B-lymphoblastic leukemia

## Abstract

Allogeneic hematopoietic stem cell transplantation (allo-HSCT) in SARS-CoV-2 positive candidates is usually delayed until the clinical resolution of the infection’s symptoms and a negative nasopharyngeal molecular test. However, prolonged SARS-CoV-2 positivity has been frequently observed in haematological malignancies, thus representing a challenge for the timing of transplant procedures. Here, we report on the case of a 34-year-old patient with recent pauci-symptomatic COVID-19 undergoing transplant for high-risk acute B-lymphoblastic leukemia before achieving viral clearance. Shortly before their scheduled allogeneic HSCT from a matched unrelated donor, the patient developed mild Omicron BA.5 infection receiving nirmatrelvir/ritonavir with fever resolution within 72 hours. Twenty-three days after COVID-19 diagnosis, because of increasing minimal residual disease values in the context of high-risk refractory leukemia and clinical resolution of SARS-2-CoV infection with reduction of viral load at surveillance nasopharyngeal swabs, it was decided not to delay further allo-HSCT. During myelo-ablative conditioning, the nasopharyngeal SARS-CoV-2 viral load increased while the patient remained asymptomatic. Consequently, two days before the transplant, intra-muscular tixagevimab/cilgavimab 300/300 mg and a 3-day course of intravenous remdesivir were administered. During the pre-engraftment phase, veno-occlusive disease (VOD) occurred at day +13, requiring defibrotide treatment to obtain a slow but complete recovery. The post-engraftment phase was characterized by mild COVID-19 at day +23 (cough, rhino-conjunctivitis, fever) that spontaneously resolved, achieving viral clearance at day +28. At day +32, she experienced grade I acute graft-versus host disease (a-GVHD, skin grade II) treated with steroids and photo-apheresis, without further complications during follow-up until day +180. Addressing the issue of allo-HSCT timing in patients recovering from SARS-CoV-2 infection with high-risk malignant diseases is challenging because of 1] the high risk of COVID-19 clinical progression, 2] the impact of transplant delay on leukemia prognosis and 3] the occurrence of endothelial complications such as VOD, a-GVHD, and transplant associated thrombotic micro-angiopathy. Our report describes the favourable outcome of allo-HSCT in a recipient with active SARS-CoV2 infection and high-risk leukemia thanks to timely anti-SARS-CoV-2 preventive therapies and prompt management of transplant-related complications.

## Introduction

Allogeneic hematopoietic stem cell transplant (allo-HSCT) recipients are at high risk of critical Coronavirus disease 2019 (COVID-19). The largest surveys to date have reported a mortality of 16-25% and the following risk factors for lower survival: age, COVID-19 within 1-year after transplant, need for intensive care (1–3). Since 2022, people with COVID-19 at risk of progression should receive antivirals (nirmatrelvir/ritonavir, remdesivir) or neutralising monoclonal antibodies (mAbs), which have been shown to significantly reduce hospitalization and death (4–6). The impact of these therapies on allo-HSCT recipients has not yet been assessed because the immunocompromised patients in registration trials have to date been a minority and the abovementioned studies (1–3) refer to first and second waves. Recently, two large analyses from EPICOVIDEHA on subjects with hematological malignancies (HM), one on Omicron infection’s outcome and another on breakthrough COVID-19 in vaccinated patients (<5% allo-HSCT recipients), reported lower mortality (<10% vs 31%) with mAbs (7, 8). Lastly, a retrospective Italian study of 328 HM patients treated for mild/moderate COVID-19 with mAbs (n=120, 37%; sotrovimab, n=73) or antivirals (n=208, 63%; nirmatrelvir/ritonavir, n=116) between March 2021 and July 2022 reported treatment failure (severe COVID-19) of 9.5%. Overall, COVID-19 associated mortality was 3.4%; however, in those who developed severe COVID-19 after early treatment, it was 26% in the Omicron wave (9). These preventive therapies are strongly recommended in HM patients, along with pre-exposure prophylaxis with tixagevimab/cilgavimab and vaccination depending on the individual patient’s type and degree of immunosuppression (10–12).

Since 2020, EBMT and ASTCT have provided recommendations to manage transplant recipients and donors, both from malignant and non-malignant indications, adopting a prioritization process to deliver HSCT with a periodic update according to pandemic evolution, therapeutic options, and prevention strategies (13–16). To date, candidates for cellular therapies who are SARS-CoV-2 positive have preferentially delayed the procedure until viral test negativity, carefully considering infection severity and the risks related to the underlying disease. Immunocompromised patients typically shed the virus ≥4 weeks; for patients with persistently low-level PCR-positivity, transplant practices are evolving, but no recommendation can be drawn. Two reports are available on performing allo-HSCT during active SARS-CoV-2 infection, both diagnosed during conditioning chemotherapy. The first patient received remdesivir and convalescent plasma, experiencing at neutrophil engraftment a cytokines release syndrome associated with mild pneumonia, successfully cured with remdesivir again, casirivimab/imdevimab, tocilizumab and methylprednisolone (17). The second patient received a transplant from a donor with asymptomatic SARS-CoV-2 infection, developing a transient respiratory failure shortly after PBSC infusion (18).

We report on the outcome of a 34-year-old female patient with B-lymphoblastic leukemia undergoing allo-HSCT shortly after COVID-19, before complete viral clearance.

## Case description

**Figure 1:**
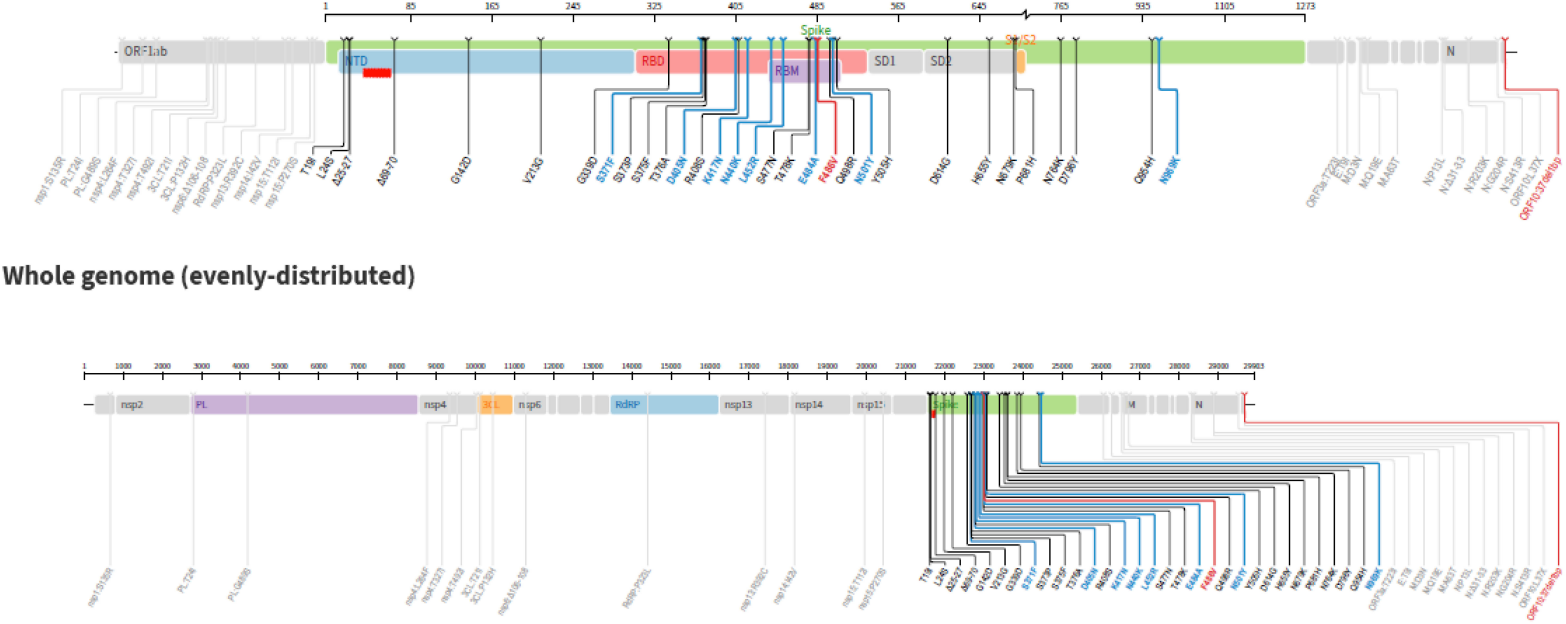
Next Generation Sequencing performed at SARS-CoV-2 infection diagnosis and repeated with the same results 3-week after nirmatrelvir/ritonavir therapy and 1-week after tixagevimab/cilgavimab and remdesivir therapy.

Despite vaccination (Moderna, booster 6 months earlier), a 34-year-old patient affected by B-lymphoblastic leukemia t(9;22)(q34;q11.2) BCR-ABL1 developed Omicron BA.5 infection while waiting for her scheduled transplant from a matched unrelated donor (MUD). She was referred to our center for allo-HSCT after treatment with Imatinib, which was withdrawn after one week for intolerance in favor of Dasatinib obtaining a first hematological complete remission with minimal residual disease (MRD) positivity. Subsequently, the patient received Ponatinib due to increased MRD levels according to GIMEMA LAL2620 (NCT04475731), but the disease relapsed. After achieving second complete morphological remission with detectable MRD after two cycles of Inotuzumab ozogamicin (IO), an allo-HSCT was scheduled. **Figure 1** displays the Next-Generation Sequencing (NGS) analysis of the virus.

The patient received prompt preventive antiviral therapy with nirmatrelvir/ritonavir, obtaining fever resolution within 72 hours and a consistent viral load reduction at nasopharyngeal swabs. Twenty-three days after COVID-19 diagnosis, based on increasing MRD values in the context of high-risk disease refractory to third generation tyrosine kinase inhibitor and clinical resolution of SARS-CoV-2 infection, it was decided not to delay further allo-HSCT. During conditioning chemotherapy [fludarabin, 12 Gy total body irradiation (TBI)], nasopharyngeal viral load increased (virus confirmed viable in cell culture), while the patient remained asymptomatic. Two days before PBSC reinfusion in July 2022, tixagevimab/cilgavimab 300/300 mg and a 3-day course of remdesivir were administered. The donor was a female 10/10 HLA-MUD, vaccinated for SARS-CoV-2. The graft-versus host disease (GVHD) prophylaxis was based on post-transplant cyclophosphamide (PT-Cy), mycophenolate mofetil, and cyclosporine A (CsA). Neutrophil engraftment was reached at +20 days after the transplant.

The pre-engraftment phase was characterized by uncomplicated febrile neutropenia without documented infections. Despite trying to minimize the risk factors for veno-occlusive disease (VOD) in the context of IO exposure and a TBI-based myeloablative-preparing regimen, administering ursodeoxycholic acid prophylaxis and using CsA rather than sirolimus as GVHD prophylaxis (19), VOD occurred at day +13 (thinning of hepatic veins, thickening of gallbladder, ascites, bilirubin peak 2.3 mg/dL, Fibroscan peak 75kPa), requiring defibrotide treatment to obtain a slow but complete resolution of this complication.

The post-engraftment phase was characterized by mild COVID-19 at day +23 (cough, rhino-conjunctivitis, fever). A spontaneous symptom resolution was observed, enabling the patient to achieve a definitive viral clearance at day +28 after transplant. At day +32, the patient experienced grade I acute GVHD (skin grade II) treated with methylprednisolone 0.5 mg/kg/daily and three cycles of weekly extracorporeal photo-apheresis as a steroid-sparing approach due to concomitant SARS-2-CoV infection, without side-effects. At day +40 after transplant with a full donor chimerism of myeloid and T-cells and in complete remission with negative MRD, the patient was discharged home. Then, a steroid dose increase was required to obtain GVHD remission, without further complications or adverse events during follow-up until day +180. Bone marrow evaluation performed at six months confirmed complete hematological remission with negative MRD, without requiring additional treatments.

**Figure 2:**
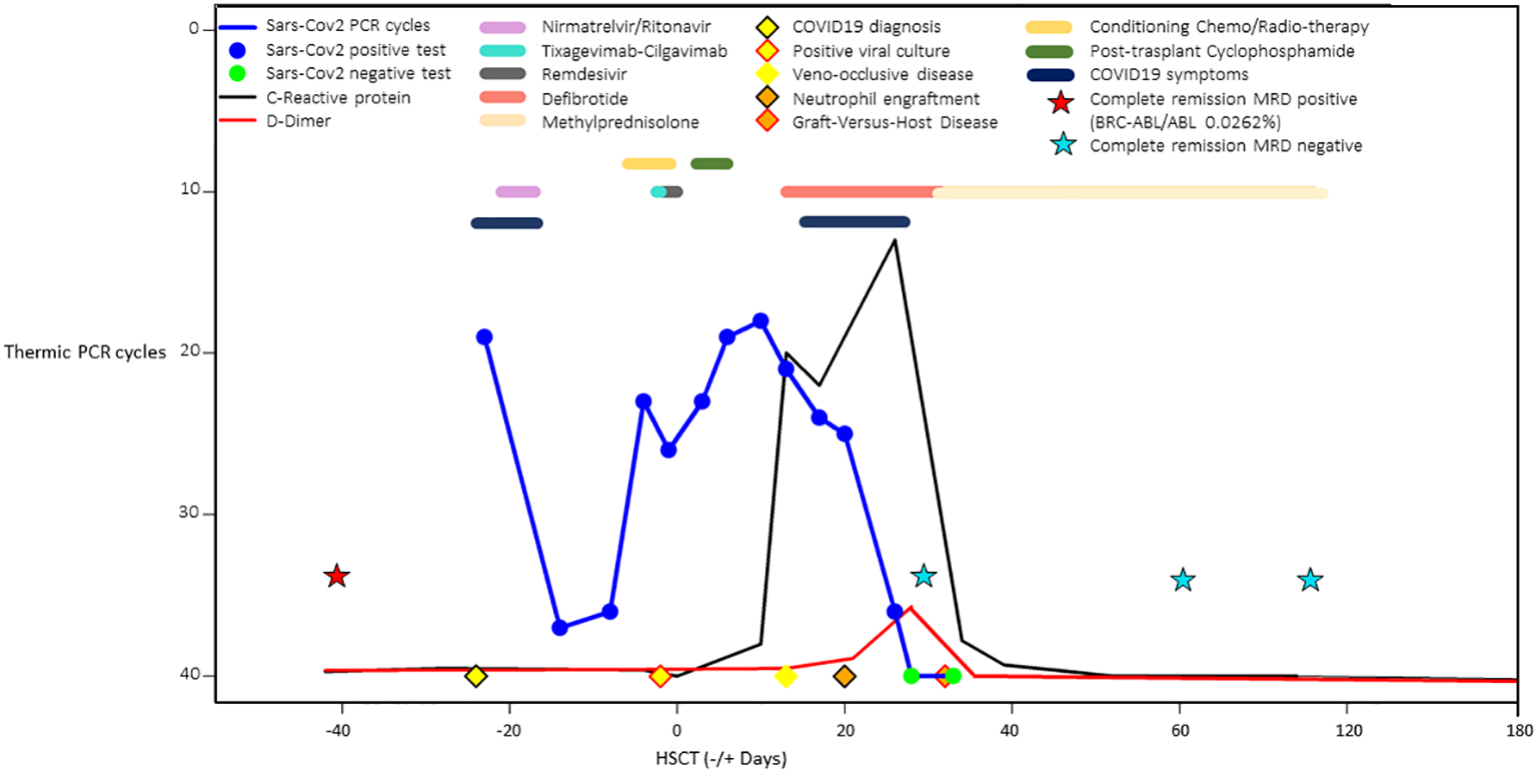
Comprehensive schematic case summary of SARS-CoV-2 nasopharyngeal viral load trends by PCR cycles, C-reactive protein and D-dimer values, COVID-19 treatments in pre and post-transplant phases, veno-occlusive disease, and acute graft-versus host disease treatments.

**Figure 2** summarizes the comprehensive clinical course of SARS-CoV-2 nasopharyngeal viral load trends by PCR cycles, C-reactive protein, D-dimer values, COVID-19 treatments in pre and post-transplant phases, veno-occlusive disease, and acute GVHD treatments.

SARS-CoV-2-specific cellular and humoral responses were also analyzed in detail and detailed methods are reported in the **Supplementary Materials** of this case study.

## SARS-CoV-2-specific cellular and humoral responses

**Figure 3:**
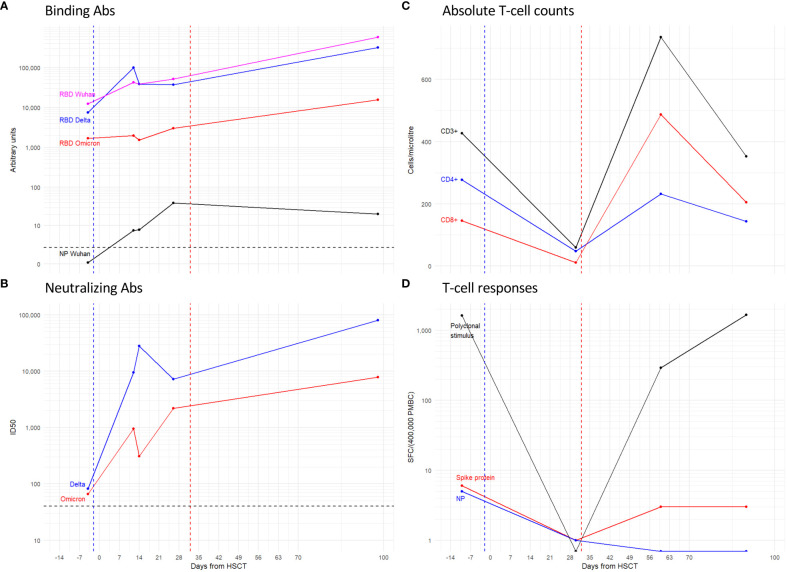
Antibody and T-cell responses to SARS-CoV-2 antigens. Line plots show: the IgG binding to the spike RBD and NP proteins **(A)**; the Nab ID50 titer **(B)** expressed as the reciprocal of the serum dilution giving 50% RLU reduction compared to control wells; the absolute counts of total CD3+ T-cells, and the CD4+ and CD8+ subsets expressed as Cells/ml **(C)**; and the magnitude of T-cell responses either polyclonal or specific for Spike and Nucleocapsid proteins expressed as specific Spot Forming Cells (SFC)\400’000 Peripheral Blood Mononuclear Cells (PBMC) **(D)**. Dashed lines: Blue coincides with the date of tixagevimab-cilgavimab administration, Red with graft-versus host disease. Black is the threshold for the positivity of the assay.

IgGs against the SARS-CoV-2 RBD were present before transplant although the omicron RBD antibody binding was 7 and 4-fold lower compared with that of the Wuhan-Hu-1 and delta RBDs, respectively. Post tixagevimab-cilgavimab administration, anti-RBD IgG levels against Wuhan and delta increased and followed a similar kinetic over time, while an omicron IgG rise lagged until day +30, which is consistent with the reduced binding by tixagevimab-cilgavimab to this VOC (**Figure 3A**). Binding IgGs against the SARS-CoV-2 nucleocapsid protein were initially negative and developed by day +37 after the positive swab.

Neutralizing Ab (NAb) against delta and omicron VOC were at low titers (1/82 and 1/66 respectively) twenty-one days after the positive swab, a time point which was, however, two days after chemotherapy (**Figure 3B**). NAb against both VOCs showed increased titers when tested at twelve days after administration of tixagevimab-cilgavimab and had similar kinetics thereafter. Overall, the neutralizing response to omicron was persistently lower than that of delta (fold range: 2-90). None of the serum samples neutralized the LV-Luc/VSV.G pseudo-viral vector, indicating that the detected neutralizing responses were specific for SARS-CoV2 (data not shown).

Both polyclonal (**Figure 3C**) and SARS-CoV-2 specific T-cell reconstitution (**Figure 3D**) were longitudinally evaluated before (-4 days) and at day +30, +60 and +90 after HSCT. Before the transplant, the patient showed a limited but detectable level of specific responses toward the Spike (6 SFC/400’000 PBMC) and the Nucleocapsid protein (5 SFC/400’000 PBMC). Early after transplant (+30 days), the frequency of both polyclonal and SARS-CoV-2 specific T-cell responses were below the limit of detection of the assay due to the low CD3+ T-cell counts (59 CD3+ T cells/µl, 48 CD4+ T cells/µl, and 11 CD8+ T cells/µl). At the following time points, the absolute counts of CD3+ T cells increased (379 and 353 CD3+ T cells/µl at day +60 and +90, respectively). Accordingly, the polyclonal T-cell response rose to 1’664 SFC/400’000 PBMC at day +90 after HSCT. However, the specific response to the Nucleocapsid protein remained negative after transplant, and the frequency of Spike-specific T-cells was assessed to be at very low levels (3 SFC/400’000 PBMC at both days +60 and +90). A progressive decrease of the viral load was observed within a few weeks after transplant and the molecular SARS-CoV-2 nasopharyngeal swab test was negative by day +28 after transplant, before the reconstitution of an adequate number of T-cells that could elicit a specific response.

## Discussion

In patients that are a candidate for transplant, either HSCT or solid organ transplant, the management of active infections shortly before the transplant is particularly challenging considering both the urgency of the transplant procedure for the risk of haematological disease progression and end-organ failures and the importance of the resolution of the infection itself to reduce the risk of severe infectious complications in the post-transplant period. The timing of allo-HSCT in candidates with recent COVID-19, especially in the 30 days before transplant, is crucial due to the risk of infection relapse and progression to life-threatening pneumonia in the pre-engraftment phase, which retains a high risk of COVID-related mortality because transplant recipients in this phase have a high immunodeficiency scoring index that is predictive of COVID-related mortality (2). Recent, evaluable clinical data focused on the choice and the best timing for transplant procedures are rare, meaning efforts to address this should be undertaken within the transplant community in the near future.

The current report has outlined the management of a patient affected by acute leukemia experiencing mild COVID-19 before starting a conditioning regimen, and being successfully treated with nirmatrelvir/ritonavir, balancing high-risk leukaemia while considering the clinical resolution of SARS-CoV-2 infection. Clinicians decided to proceed with conditioning chemotherapy and transplantation, even in the absence of viral clearance indicated by molecular nasopharyngeal swab. As anticipated, the patient had an asymptomatic recurrence of Omicron BA.5 infection during conditioning chemotherapy, meaning a double combination therapy was instituted (remdesivir plus tixagevimab-cilgavimab) to contain the risk of progression to severe COVID-19 in the pre-engraftment phase. At the end of conditioning and before starting GVHD prophylaxis, a double dose of tixagevimab-cilgavimab 300/300 mg was administered to obtain neutralization activity against Omicron BA.5, which is less susceptible to tixagevimab-cilgavimab than previous circulating SARS-CoV-2 variants, along with a 3-day course of remdesivir, not previously received by the patient. We decided to administer such therapies early while waiting for the results of the NGS analysis, to limit drug interactions and eventual toxicities. Thanks to those therapeutic interventions and close clinical monitoring, the patient experienced only mild COVID-related symptoms concomitantly to neutrophil engraftment, without progression to interstitial pneumonia. Considering the circulation of immune-escape variants less efficiently neutralized by mAbs (20) and the risk of promoting mutated viruses in immunocompromised hosts with protracted infection, this report suggests the potential role of NGS analysis to institute effective treatments in a timely manner. However, a shorter turn-around time for the results report is needed for its implementation into clinical daily practice. In this patient, the NGS analysis later revealed the presence of E484A mutation in the Spike protein that is characteristic of all Omicron variants, featuring resistance to bamlanivimab/etesevimab, casirivimab/imdevimab, and tixagevimab, and T21I mutation in the Protease-inhibitor protein associated with *in-vitro* low-level resistance to nirmatrelvir (21). This suggests the value of using mAbs in combination with one or even more than one antiviral drug (i.e. remdesivir plus nirmatrelvir) to achieve a higher probability of clinical cure and viral clearance in selected settings, such as HM patients closer to transplant (22, 23).

Although preventive therapies play a critical role in mitigating the risk of severe COVID-19 in the pre-transplant and pre-engraftment phases, allowing the transplant procedure in urgent cases of high-risk HM to take place, both recipient revaccination and donor vaccination are of paramount importance, with the latter promoting recipient’s humoral response to early revaccination (24).

The other major issue of performing allo-HSCT during or shortly after COVID-19 is the eventual occurrence of post-transplant endothelial complications, which may occur due to the potential pathogenesis overlap between COVID-associated endothelitis, VOD, transplant associated-thrombotic micro-angiopathy (TA-TMA), and GVHD. In this regard, Niederwieser et al. discuss fourteen patients who received allo-HSCT after recovering from COVID-19, with 21% of patients developing VOD and/or TA-TMA, which was successfully treated with defibrotide, prednisone, or eculizumab; interestingly, all patients were female and received a mismatched transplant from male donors (25).

The current case confirms the potential risk of post-transplant endothelial complications in relation to active SARS-CoV-2 infection since both VOD and acute GVHD were reported in the patient; however, it also outlines the presence of several risk factors predisposing to VOD in particular. The timely administration of defibrotide and a tailored GVHD treatment succeeded in obtaining the resolution of both complications.

Data on viral specific immune-reconstitution has only been reported in this clinical report and have not been available in routine clinical practice to date. The SARS-CoV-2 specific T-cell response of this patient before conditioning was low; after transplant, it was even lower, which contrasts with a previous report by Pourhassan (17). This observation probably resulted from both the *in-vivo* T-cell depletion with PT-Cy and the reasonably fast viral clearance achieved at day +28 after transplant, thus limiting antigen exposure and the subsequent development of SARS-CoV-2-specific response despite good functional T-cell recovery observed for polyclonal stimuli. In the early phase after PT-Cy-based transplants, the rate of viral infections is high and the emergence of viral-specific T-cells in primary immunological responses has been shown to be largely sustained by antigen encounter on host-infected cells rather than by cross-priming/presentation by non-infected donor-derived antigen-presenting cells (26, 27).

The immune protection displayed by our patient in the pre-engraftment phase was predominantly conferred by tixagevimab-cilgavimab, even though a contribution of natural humoral response was observed with the presence of antibodies against nucleocapside at day +37. During the transplant planning phase, the choice of a vaccinated donor may be beneficial, potentially mitigating the risk of progression to severe COVID-19. Moreover, we suggest that the management of SARS-CoV-2 infection during the pre-engraftment phase should favour a timely combination therapy [two antivirals (remdesivir plus nirmatrelvir) in association or not with mAbs depending on SARS-CoV-2 variant].

In conclusion, addressing the issue of allo-HSCT timing in patients recovering from SARS-CoV-2 infection with high-risk malignant diseases is challenging because of the high risk of the clinical progression of COVID-19, the impact of transplant delay on leukemia prognosis, and the potential higher incidence of endothelial complications. Although it is only a single case, this report suggests that successful allo-HSCT in patients with active COVID-19 is feasible with prompt SARS-CoV-2-specific preventive therapies. Recognizing the importance of early SARS-CoV-2-specific T-cell expansion approaches that can enhance specific immune recovery, such as adoptive cellular therapy (28), should be considered for patient management, especially in mismatched transplants with *in-vivo* T-cell repletion.

## Data availability statement

The raw data supporting the conclusions of this article will be made available by the authors, without undue reservation.

## Ethics statement

The studies involving human participants were reviewed and approved by San Raffaele Hospital Ethics Committee. The patients/participants provided their written informed consent to participate in this study.

## Author contributions

CO and RG coordinated the collection of data. CO, RG, AnA, and GO collected and assembled the data, and interpreted the analysis. CO wrote the first draft of the manuscript. RG was involved in reviewing and editing the manuscript. NC and MS performed Next-Generation Sequencing analysis. MN, ET, VB, SD, and IM performed SARS-2-CoV ELISpot analysis and humoral tests. All authors critically revised, edited, and approved the manuscript.
