# Supplementary material for: Case Report: Favorable outcome of allogeneic hematopoietic stem cell transplantation in SARSCoV2 positive recipient, risk-benefit balance between infection and leukemia
Source: Front Immunol. 2023 May 23;14:1184956. doi: 10.3389/fimmu.2023.1184956 (PMC10242072; doi:10.3389/fimmu.2023.1184956)
Supplement: Supplementary file 1 [file DataSheet_1.docx]

**Supplementary materials**

**SARS-CoV-2 specific T-cell response**

Frequencies of IFN-γ-producing SARS-CoV-2-specific T cells were evaluated by means of IFN-γ enzyme-linked immunospot assay (ELISpot, Mabtech ELISpot kit) on Peripheral Blood Mononuclear Cells (PBMC). Cells were kept in culture for two hours in IMDM (Lonza) supplemented with 10% Human Serum, Glutamine (1%), and Penicillin/Streptomycin (1%) in the presence of low doses of recombinant human IL-2 (rhIL-2, 20 UI/ml, Novartis). Cells were then washed to eliminate the rhIL-2. PBMC (400’000/well) were stimulated for 16-20 hours with two libraries of overlapping peptides spanning the SARS-CoV-2 Spike and the Nucleocapsid proteins: PepTivator SARS-CoV-2 S and N, respectively (1 μg of each peptide/ml; Miltenyi). Anti-CD3 monoclonal antibodies were used as positive control and irrelevant peptides as negative control (pool of peptides covering the ovalbumin sequence, 1 μg of each peptide/ml; Miltenyi). Anti-CD28 monoclonal antibodies (1 μg/ml; BD) were added in each condition to provide costimulatory signals and increase T-cell stimulation. Each condition was run in duplicate. Spot-forming cells (SFC) were quantified by the ImmunoCapture 7.0 software (TLC ELISpot Reader). Unstimulated T cells and the negative control were subtracted. Results were expressed as specific SFC/400’000 PBMC.

**Absolute counts of lymphocyte subsets**

The polyclonal Immune Reconstitution (IR) was evaluated by flow cytometry on whole blood samples, using a lyse-no-wash technique and a panel of directly conjugated antibodies specific for CD45, CD3, CD4, CD8, CD19, CD16, CD56, CD25 and CD127, according to the International Society for Cell Therapy (ISCT) immunological gating protocol^1^. The single platform method was used to determine absolute counts, employing fluorospheres (Flow-Count™, Beckman Coulter). Cells were acquired with a Navios cytometer (Beckman Coulter).

**Lentiviral Vector-Based SARS-CoV-2 Neutralization Assay**

Lentiviral vector pseudotypes (LV-luc) expressing delta (B.1.617.2) and omicron (B.1.1.529) spike and luciferase^2^ were titered on VeroE6 cells (African green monkeys, epithelial kidney) performing five replicates of serial 2-fold dilutions in growth medium in 96-well culture plates. Dilution providing 150,000–200,000 relative luciferase units (RLU) were used in the neutralization assay, as previously described^3^. Briefly, heat-inactivated serum serial threefold dilutions starting from the 1/40 dilution were incubated in duplicate with the LV-Luc for 30 min at 37 °C in 96-well plates, and thereafter added to VeroE6 cells at a density of 20,000 cells/well. After 48 h, luciferase expression was determined with a luciferase assay system (Bright-Glo, Promega) and measured in a Mitras luminometer (Berthold, Germany). The 50% inhibitory serum dilution (ID50) was calculated with a linear interpolation method using the mean of the duplicates^3^. Neutralization was expressed as the reciprocal of the serum dilution giving 50% inhibition of RLU compared to the mean of the virus control wells. An ID50 below 1/40 serum dilution was considered negative and a value of 10 ascribed for statistical analysis. To exclude any unspecific inhibition each sample was tested in duplicate at 1/40 dilution with LV-Luc/VSV.G in the neutralization assay, as described above.

**IgG Binding Antibody Luciferase Immunoprecipitation System (LIPS) Assay**

IgG binding to SARS-CoV-2 Wuhan-Hu-1, delta (B.1.617.2) and omicron (B.1.1.529) spike RBD domains, and to Wuhan-Hu-1 nucleocapsid protein (NP) was measured by LIPS^4^ using recombinant nanoluciferase tagged antigens, as previously described^5-6^. Briefly, each recombinant antigen was expressed by transient transfection into Expi293F™ cells (Expi293™ Expression System, Thermo Fisher Scientific Life Technologies, Carlsbad, CA, USA) and incubated in liquid phase with test serum (1ul) for 2 h. Immune-complexes were then captured with rProtein A-sepharose and centrifugation. After 5 washes the sepharose pellets the presence of IgG bound antigen was determined by measuring the recovered luciferase activity in a Berthold Centro XS3 luminometer (Berthold Technologies GmbH & Co. KG, Bad Wildbad, Germany). Raw data was converted into arbitrary units (AU) using a local positive index serum that exhibited closely similar binding to all of the variants of concern (VOCs).

**Supplemental References**

1. Mfarrej B, Gaude J, Couquiaud J, *et al*. Validation of a flow cytometry-based method to quantify viable lymphocyte subtypes in fresh and cryopreserved hematopoietic cellular products. *Cytotherapy* 2021; 23(1):77-87.
2. Dispinseri S, Marzinotto I, Brigatti C, *et al*. Seasonal Betacoronavirus Antibodies' Expansion Post-BNT161b2 Vaccination Associates with Reduced SARS-CoV-2 VoC Neutralization. *J Clin Immunol* 2022; 42(3):448-458.
3. [Fenyö](https://pubmed.ncbi.nlm.nih.gov/?sort=date&term=Feny%C3%B6+EM&cauthor_id=19229336) EM, [Heath](https://pubmed.ncbi.nlm.nih.gov/?sort=date&term=Heath+A&cauthor_id=19229336) A, [Dispinseri](https://pubmed.ncbi.nlm.nih.gov/?sort=date&term=Dispinseri+S&cauthor_id=19229336) S, *et al*. International network for comparison of HIV neutralization assays: the NeutNet report. *PLoSONE* 2009;4(2):e4505.
4. Burbelo PD, Goldman R, Mattson TL. A simplified immunoprecipitation method for quantitatively measuring antibody responses in clinical sera samples by using mammalian-produced *Renilla* luciferase-antigen fusion proteins. *BMC Biotechnol* 2005;18(5):22.
5. [Secchi M](javascript:void(0);), [Bazzigaluppi E, Brigatti](javascript:void(0);) C, *et al*. COVID-19 survival associates with the immunoglobulin response to the SARS-CoV-2 spike receptor binding domain. *J Clin Invest*2020;130(12):6366-6378.
6. Lind A, Marzinotto I, Brigatti C, *et al*. A/H1N1 hemagglutinin antibodies show comparable affinity in vaccine-related Narcolepsy type 1 and control and are unlikely to contribute to pathogenesis. *Sci Rep* 2021;11(1):4063.
